# Climate and human health: a review of publication trends in the International Journal of Biometeorology

**DOI:** 10.1007/s00484-023-02466-8

**Published:** 2023-05-02

**Authors:** Ogone Motlogeloa, Jennifer M. Fitchett

**Affiliations:** School of Geography, Archaeology and Environmental Studies, University of the Witwatersrand, Johannesburg, South Africa

**Keywords:** Health biometeorology, Climate and health, Diseases, Meteorological variables, Publications, Human

## Abstract

The climate-health nexus is well documented in the field of biometeorology. Since its inception, Biometeorology has in many ways become the umbrella under which much of this collaborative research has been conducted. Whilst a range of review papers have considered the development of biometeorological research and its coverage in this journal, and a few have reviewed the literature on specific diseases, none have focused on the sub-field of climate and health as a whole. Since its first issue in 1957, the *International Journal of Biometeorology* has published a total of 2183 papers that broadly consider human health and its relationship with climate. In this review, we identify a total of 180 (8.3%, *n* = 2183) of these papers that specifically focus on the intersection between meteorological variables and specific, named diagnosable diseases, and explore the publication trends thereof. The number of publications on climate and health in the journal increases considerably since 2011. The largest number of publications on the topic was in 2017 (18) followed by 2021 (17). Of the 180 studies conducted, respiratory diseases accounted for 37.2% of the publications, cardiovascular disease 17%, and cerebrovascular disease 11.1%. The literature on climate and health in the journal is dominated by studies from the global North, with a particular focus on Asia and Europe. Only 2.2% and 8.3% of these studies explore empirical evidence from the African continent and South America respectively. These findings highlight the importance of continued research on climate and human health, especially in low- and lower-middle-income countries, the populations of which are more vulnerable to climate-sensitive illnesses.

**Supplementary Information:**

The online version contains supplementary material available at 10.1007/s00484-023-02466-8.

## Introduction

The history and the development of the International Society of Biometeorology are well documented in the *International Journal of Biometeorology* (Tromp 1957; International Society of Biometeorology 1975, 1981; Keatley 2017). Since its inception, the *International Journal of Biometeorology* has not only been true to its international scope, but also to the variety of studies it has published that span across various fields of research (Sheridan and Allen 2017; Fitchett 2021). The role of climate on health has been known for millennia, yet the deliberate study of the aetiology and statistical analysis have been more recent, and a key component of biometeorology, since the inception of the society and the first issue of the journal (Tromp 1956). Increased incidence of respiratory and cardiovascular disease (CDC 2022), injuries and early deaths from extreme weather events (CDC 2022), changes in the prevalence and geographic distribution of food- and water-borne illnesses and other infectious diseases, and threats to mental health are some of the health effects that are worsened by a change in climate (CDC 2022; Krüger et al. 2022). Changes in climate and climatic variability, particularly changes in the frequency, intensity, and distribution of weather extremes, affect the environment that provides us with clean air, food, water, shelter, and security (Wu et al. 2016; Wang et al. 2017). Given that the impacts of climate change are projected to increase over the next century, existing climate-sensitive health threats will intensify and new health threats may emerge particularly in regions situated in the Southern Hemisphere and regions in Europe with an ageing population (Caini et al. 2018). The studies published in the *International Journal of Biometeorology* on the impact of various meteorological variables on disease are important as their findings not only help classify the severity of the situation but also assist in better understanding how climatically sensitive these diseases are (Hewitt and Griggs 2004). This journal serves as an important platform, encouraging from its inception the collaboration between climatologists, health practitioners, and a range of other interdisciplinary actors (Sheridan and Allen 2017). This subsequently allows for the development of effective methods for minimizing the effects of climate change on health (Tromp 1957).

Three review papers on health biometeorology have been published in the *International Journal of Biometeorology*. Cheng et al. (2019) reviewed the impacts of exposure to ambient temperature on the burden of diseases; Hossain et al. (2019) examined the relationship between sociodemographic factors, climate, and respiratory tract infections in the literature; and Jahan et al. (2020) published a review on the relationship between schizophrenia and seasonality. These papers made important contributions in synthesizing the research for specific medical conditions, However, this study presents the first systematic review that considers the subdiscipline of health biometeorology as a whole, reflecting on the geographic distribution of studies, the diseases investigated, the specific meteorological variables investigated, and the growth in this thematic area within the journal from 1957 to April 2022. This review paper contributes to the ongoing reflective and reflexive efforts of this journal and relatively new discipline in tracking the growth and impact of research. It is also valuable in evaluating whether the trends in the literature align with the key global challenges faced at present.

## The establishment of health biometeorology research

The meteorologist Franz Linke (1878–1944) formalized the academic theme of medical meteorology (Tromp 1963). Rather than concentrating on independent meteorological components, Linke examined the connection between complexes of weather conditions factors, for example, such as frontal passages and air masses with biological events (Tromp 1977). To acquire vital information, Linke established bioclimatological stations across Germany and started to give clinical meteorological gauges in light of measurable meteorological examinations (Tromp 1967, 1977). To convey the outcomes, Linke established, along with Austrian partners, the main journal committed to bioclimatology, the ‘Bioklimatische Beiblätter’ as an enhancement to the Austrian ‘Meteorologische Zeitschrift’ (de Rudder 1931; International Society of Biometeorology and Bioclimatology 1957). The journal integrated a few previously divergent disciplines, including physics, medicine, botany, and geography, that were relevant to the interdisciplinary study of biology and the climate (International Society of Biometeorology and Bioclimatology 1957). Parallel to the holistic movement, a few German meteorologists and doctors concentrated on the Hippocratic theories on the connection between weather and health through unbiased and more logical methodologies (Tromp 1963, 1966). Regardless of the revolution in medication that brought about germ theory, bacteriology, and parasitology as focal ideas in medication, more established Hippocratic convictions about the natural reasons for sickness continued (Lieth 1988). These thoughts were communicated in various structures, subject to the nearby setting and the expectations of the people who embraced them (Tromp 1966; Brezina 1938).

Holistic values underscored an organic perspective on man as a patient, creating opportunities for natural healing, for example, climatotherapy (Bynum 1994). The theoretical, order Hippocratic idea that climatic fluctuation caused sickness was embraced in the logically arranged approaches of Petersen (1935), de Rudder (1931), and others, which became known as ‘bioclimatology’ or ‘biometeorology’. Considering the longing to understand the link between man and his environment, it is not surprising that this concept was selected amongst the many other Hippocratic theories (Weihe 1967). To follow the onset of disease, which is a diversion from the healthy state, it was sensible to look for related changes in the environment, the climate, or the weather (Tromp 1977). It was unanticipated that during the 1920s meteorologists would have made great leaps forward in how they might interpret changes in the climate; thus, scientists such as de Rudder (1931) and Petersen (1935) began to concentrate on how the onset of diseases is related to large scale atmospheric movements (de Rudder 1931; Petersen 1935). However, It is less certain how more subtle environmental factors play a role in the origin of diseases (Wu et al. 2016).

Biometeorology is the field that studies relationships between living organisms and their surroundings, with its sub-branch of human biometeorology dedicated to issues concerning human health (Tromp 1977). These fields are multidisciplinary, combining biology or medicine with meteorology and climatology (Folk 2013). Around 1950, biometeorological topics were studied by dispersed and isolated individual scientists with various backgrounds (McGregor 2012). A need was felt to organize these efforts into a new discipline with its institutions in the form of a society, a journal, dedicated conferences, and study groups (Folk 2013). The discipline was formalized in the 1950s with the establishment of the *International Society of Biometeorology* and its flagship journal in 1956 and 1957 respectively (Folk 2013). Studies such as these bring us to the modern-day and subsequently increase in recognition of the need to statistically engage in the relationship between climate and human health. For this reason, this study focuses specifically on empirical papers that use a statistical approach with meteorological data and case data.

## Methods

**Fig. 1:**
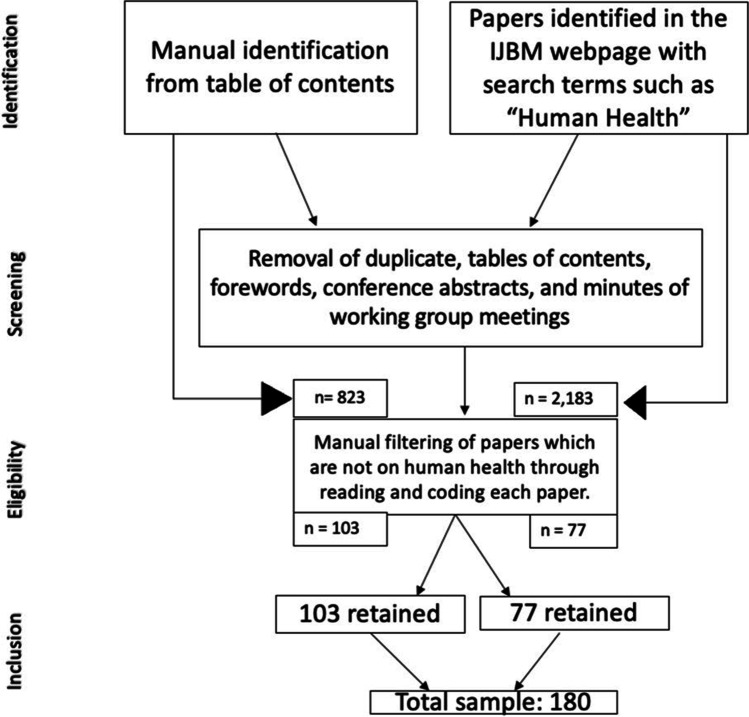
Flow diagram of the process of article identification, screening, eligibility, and inclusion in this review

The methodology employed in this review is adapted from previous discipline-specific reviews in the *International Journal of Biometeorology*, which consider only those papers published within the journal (Beggs et al. 2017; Donnelly and Yu 2017; Hondula et al. 2017; Sheridan and Allen 2017). The SpringerLink search function on the *International Journal of Biometeorology* webpage was used in the data acquisition process of this review, from which search words were applied across all published papers within the journal. An initial search was conducted for research papers using the phrase ‘human health’, returning all papers in which both ‘human’ and ‘health’ are mentioned at least once in the title, abstract, and main text of the manuscript. This yielded a total of 2183 papers (Fig. 1).

Search results that included conference abstracts, review papers, minutes from working group papers, corrections, and forewords of issues were excluded in the first round of screening. At this point, 2183 studies broadly considering climate and health through empirical research were identified (Fig. 1). A descriptive analysis of the thematic areas of these studies was performed, which included a manual review of each paper to ascertain whether the analysis related directly to health and climate. As this review paper specifically focuses on the intersection between specific, diagnosable diseases and meteorological variables through the use of statistical methodologies such as regressions, time series analysis, and distributed lag nonlinear models, generalized additive models with Poisson distribution, the second round of manual screening excluded all papers on broader health issues such as thermal comfort, thermal stress, fertility, and balneotherapy. Thermal comfort and stress have been the focus of prior reviews (e.g., Vanos et al. 2010; Baruti et al. 2019), and whilst certain diseases are precipitated and exacerbated by thermal stress, it is not independently considered a disease. Likewise, whilst issues of fertility may be exacerbated or triggered by the climate, this review only considers cases where specific diagnosed diseases are considered. Although balneotherapy was one of the key areas of interest in early health biometeorology (McGregor 2012), it is focused largely on general well-being rather than a specific disease, and so these themes have likewise been excluded even where balneotherapy is considered in relation to a diagnosed disease if meteorological factors have not been considered.

Of the 2183 studies, initially identified, only 77 were retained following this exclusion process (Fig. 1). This was then proceeded by manually reading through the table of contents for each issue of the journal since its inception, identifying all papers related to a specific, diagnosable disease, and meteorological variables. The sample was thereafter narrowed to papers that applied statistical techniques to empirically test the relationship between specific, measured meteorological variables, and diagnosed diseases solely. A total of 823 papers were initially identified through this process (Fig. 1). After careful consideration of each of the 823 papers, based on the exclusion criteria that were applied to the papers identified on the SpringerLink search, of these, 720 papers were excluded (Fig. 1). This brought the total down to 103 papers (Fig. 1). These papers were then manually read to determine whether they were indeed studies related to a specific disease and meteorological variables. Those that met the inclusion criteria were then captured in the database, according to the authors, year of publication, country, continent, meteorological variable, disease, and whether or not there was statistical significance. The two databases were then combined, and duplicates were removed. After careful inspection a final total of 180 papers remained in the database (Fig. 1). This represents 4.13% of the 4357 published papers in the *International Journal of Biometeorology* as of April 2022.

The 180 papers were manually coded, extracting and recording details on the diseases that were examined, in which country, with which meteorological variables, and in which year, to establish any spatial, thematic, and temporal trends in health biometeorology publications in the journal. The results of these findings have been displayed in tables, maps, and graphs, similar to those of Sheridan and Allen (2017) and Fitchett (2021). Additionally, a bibliometric network visualization was produced using VOSviewer, through the manual compilation of the same list of 180 papers. VOSViewer then generated a network visualization of the clustered articles. The nodes in the network are coloured based on their cluster membership, and the edges indicate the strength of the relationships between the nodes. The size of the nodes represents the number of articles associated with that membership. This review paper aims to track the bibliometric trends in the literature, and therefore it does not present a critique of the papers or review their approach or findings; rather, it aims to explore the publication trends within this theme of the journal.

## Results and discussion

### Publication trends

**Fig. 2:**
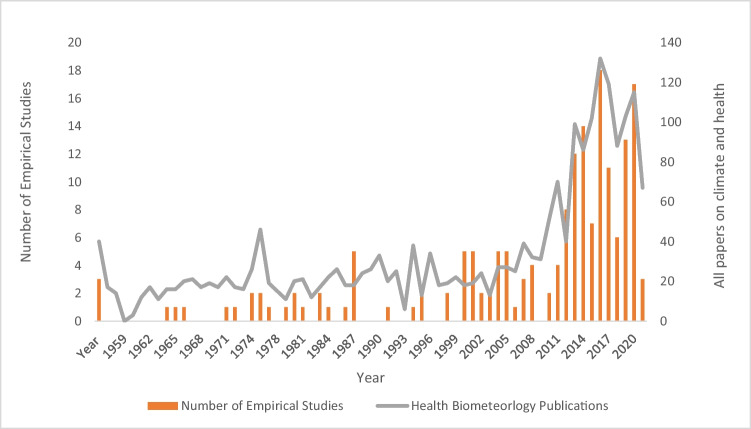
Number of empirical studies and papers related to climate and human health published per year in the *International Journal of Biometeorology*

A total of 2183 papers in the *International Journal of Biometeorology* published between 1957 and 2022 make mention of ‘human health’ at some point in the title, abstract, or manuscript, representing a substantial 50.2% of papers in the journal. Papers that explored thermal comfort account for 32.4% (*n* = 708) of this total, whilst publications assessing balneotherapy account for 5.4% (*n* = 117). There have been special issues in the journal dedicated to Balneology (Volume 64, Issue 6, June 2020) and the Universal Thermal Climate Index (Volume 56, Issue 3, May 2012 and Volume 65, Issue 9, September 2021). There is an overall increase in publications on climate and human health in the journal over the period 1957–2012. This coincides with changes in the total number of papers in the journal (Sheridan and Allen 2017). The increased number of issues per volume during the 2000s, from four issues a year until 2004, consist of 10–18 papers to six issues until 2012, and thereafter 12 issues which coincide with the months of the year (Fig. 2). These increases in health publications coincide with the inception of the constitution of the climate and human health commission (CHC) proposed in 2005 at the International Congress on Biometeorology in Germany (Fdez-Arroyabe and Robau 2017). Since its inception, the CHC has held a total of six meetings to date in 2006, 2007, 2008, 2010, 2011, and 2014 (Fdez-Arroyabe and Robau 2017). These patterns are also consistent with the temporal patterns in the total number of papers on human health in the journal over this period, attributed to advancements in technologies, moving to online publications and reviews, and the move to the Editorial Manager system in 2006 (Sheridan and Allen 2017).

The 180 papers that empirically explore the statistical relationship between meteorological variables and diagnosed disease case numbers, and which form the focus of this review (Table 1), represent 4.1% of the total 4350 papers published in the *International Journal of Biometeorology* since its inception in 1957 and 8.26% of the total 2183 papers that mention ‘human health’.

**Table 1:** Papers on the intersection of specific diagnosable diseases and meteorological variables in the *International Journal of Biometeorology* 1957–2022

Author	Year	Disease	Country	Meteorological variable	Statistical significance
Canto Borreguero	1957	Allergic diseases	Spain	Weather	Yes
Tromp	1957	Bronchial asthma and mental diseases	Netherlands	Weather	Yes
Whiten	1957	Rheumatism	Great Britain	Weather	Yes
Derrick	1965	Asthma	Australia	Weather	Yes
Tromp and Bouma	1966	Arthritic pain	Netherlands	Weather	Yes
Paulus and Smith	1967	Allergic bronchial asthma	USA	Weather and air pollutants	Yes
Hansen and Pedersen	1972	Perforated duodenal ulcer	Denmark	Barometric pressure	No
Gomersall and Stuart	1973	Migraines	Scotland	Temperature	Yes
Bodhe and Mokashi	1975	Peptic ulcer	India	Relative humidity	Yes
Specht et al	1975	Asthma	Australia	Wind	No
Von Deschwanden and Jungmann	1975	Mental illness (schizophrenia and depression); hay fever; asthma	Germany; London; USA	Temperature	Yes
Shiffman et al	1976	Intestinal disease (diarrhoea)	Guatemala	Rainfall and temperature	Yes
Fleischer and Asnani	1978	Asthma	Kenya	Rainfall and temperature	Yes
Schulman et al	1980	Headaches	USA	Barometric pressure	No
Osterman et al	1981	Headaches	Sweden	Temperature	Yes
Goldstein	1981	Asthma	New York City and New Orleans	Weather	Yes
Deacon and Williams	1982	Sudden infant death syndrome	8 metropolitan communities	Temperature and rainfall	Yes
Formiconi and Tagliaferri	1984	Urinary stone colic	Italy	Temperature, rainfall and barometric pressure	Yes
Mukammal et al	1984	Cardiovascular disease (ischaemic heart disease)	Canada	Temperature	Yes
Bellossi et al	1985	Myocardial infarction	France	Relative humidity	No
Fujita	1987	Urinary stone colic	Japan	Temperature and barometric pressure	Yes
Fletcher	1988	Föhn illness	Canada	Temperature and wind	Yes
Garvey et al	1998	Depression	USA	Temperature	No
Mannino and Washburn	1989	Cardiovascular disease	USA	Temperature and rainfall	Yes
Morton	1988	Headaches	Canada	Relative humidity	Yes
Suzuki et al	1988	Asthma	Japan	Temperature and barometric pressure	Yes
Collier	1992	Meningococcal meningitis	United Kingdom	Temperature	Yes
Ohtsuka et al	1996	Diabetes mellitus	Japan	Temperature	Yes
Halpern et al	1995	Foetal chromosome abnormalities (trisomy-21)	Israel	Solar activity	No
Thompson et al	1996	Myocardial infarction	England	Temperature and relative humidity	Yes
Aikman	1997	Arthritis (rheumatoid and osteoarthritis)	Australia	Temperature, rainfall and barometric pressure, relative humidity	Yes
McGregor et al	1999	Respiratory disease	England	Barometric pressure	Yes
Rutherford et al	1999	Asthma	Australia	Wind	Yes
Bentham and Langford	2001	Food poisoning	England and Wales	Temperature	Yes
Laaidi	2001	Pollinosis	France	Wind, relative humidity, rainfall, and temperature	Yes
Schreiber	2001	Dengue	Puerto Rico	Temperature and relative humidity	Yes
Vaitl et al	2001	Headaches and migraines	Germany	Temperature	Yes
Vocks et al	2001	Atopic eczema	Switzerland	Temperature	Yes
Gagnon et al	2002	Malaria	Colombia, Ecuador, French Guiana, Guyana, Peru, Suriname, and Venezuela	Temperature and rainfall	Yes
Makie et al	2002	Cerebrovascular disease, respiratory disease, digestive diseases	Japan	Temperature and barometric pressure	Yes
Rusticucci et al	2002	Cerebrovascular disease, respiratory disease, digestive diseases, muscle pains, skin and allergies, neurological diseases, and psychopathological disorders	Argentina	Temperature and precipitation	Yes
Schlink et al	2002	Respiratory disease	Germany	Solar radiation, temperature, relative humidity	Yes
Wang et al	2002	Stroke	Japan	Temperature	No
Kolivras and Comrie	2003	Coccidioidomycosis (valley fever)	USA	Temperature and rainfall	Yes
Tobías et al	2013	Asthma	Spain	Wind	No
Ebi et al	2004	Cardiovascular diseases (myocardial infarction, angina pectoris, congestive heart failure) and stroke	USA	Temperature and rainfall	Yes
Ohwaki et al	2004	Hypertensive intracerebral haemorrhage	Japan	Temperature	No
Bulbena et al	2005	Anxiety	Spain	Wind, rainfall, temperature, relative humidity, and solar radiation	Yes
Gyan et al	2005	Asthma	Trinidad	Wind	Yes
Kovats et al	2005	Campylobacter	Canada, Scotland, Australia, Malta, Denmark, Spain, Czech Republic, Estonia, Greece, Ireland	Temperature	Yes
Stoupel et al	2005	Down syndrome	Israel	Solar radiation	Yes
Villeneuve et al	2005	Asthma	Canada	Relative humidity, temperature, rainfall	Yes
Fleury et al	2006	Bacterial enteric infection	Canada	Temperature	Yes
Hrushesky et al	2006	Uterine cervical human papilloma virus	Holland	Temperature, rainfall, relative humidity	Yes
Nastos and Matzarakis	2006	Respiratory infection	Greece	Temperature, relative humidity, sunshine hours, wind, and barometric pressure	Yes
Ohshige et al	2006	Stroke	Japan	Temperature, relative humidity, and barometric pressure	Yes
Zender and Talamantes	2006	Coccidioidomycosis (valley fever)	USA	Rainfall and temperature	Yes
Liang et al	2008	Acute coronary syndrome	Taiwan	Temperature	Yes
Nakaguchi et al	2008	Intracerebral haemorrhage	Japan	Barometric pressure, temperature, relative humidity, rainfall, and wind	Yes
Prospero et al	2008	Asthma	Caribbean	Wind	No
Suárez-Varela et al	2008	Atopic eczema	Spain	Rainfall, temperature, and relative humidity	Yes
García-Marcos et al	2009	Asthma	Spain	Relative humidity and temperature	Yes
Liang et al	2009	Chronic obstructive pulmonary disease	Taiwan	Temperature	Yes
Styra et al	2009	Cardiovascular disease	Lithuania	Barometric pressure	No
Wang et al	2009	Stroke	Australia	Temperature	Yes
Arnedo-Pena et al	2011	Asthma and allergies	Spain	Sunshine hours	Yes
Azevedo et al	2011	Respiratory and cardiovascular diseases	Portugal	Sunshine hours	Yes
Omonijo et al	2012	Measles	Nigeria	Temperature	Yes
Goggins et al	2012	Stroke	China	Temperature, humidity, sunshine hours, precipitation, and barometric pressure	Yes
Ferrari et al	2012	Obstructive pulmonary disease	Germany	Relative humidity, solar radiation, temperature	Yes
Coelho and Massad	2012	Leptospirosis	Brazil	Weather temperature and rainfall	Yes
Alexander	2013	Heart disease, arrhythmia, heart failure, cardiopulmonary arrest, angina, pectoris, psychiatric diseases, stroke, transient ischemic attack	Argentina	Temperature and rainfall	Yes
McWilliams et al	2013	Psychotic illnesses	Ireland	Wind, rainfall, temperature, relative humidity, solar radiation, and barometric pressure	No
Scheidt et al	2013	Migraines	Germany	Temperature	Yes
Bakal et al	2013	Acute coronary syndrome	Global	Temperature and relative humidity	Yes
Arnedo-Pena et al	2013	Asthma	Western Europe	Temperature, rainfall, sunshine hours, and relative humidity	Yes
Lim et al	2013	Cardiovascular disease	Korea	Temperature	Yes
Soyiri et al	2013	Asthma	England	Temperature	No
Akinbobola and Omotosho	2013	Malaria	North Central Nigeria	Temperature, rainfall, and relative humidity	Yes
Yackerson et al	2014	Schizophrenia	Israel	Wind and barometric pressure	Yes
de Weger et al	2014	Allergic rhinitis	Netherlands	Temperature	Yes
Wanka et al	2014	Respiratory disease	Germany	Temperature and relative humidity	No
Smedslund et al	2014	Fibromyalgia pain	Norway	Barometric pressure, temperature, and relative humidity	Yes
Shaposhnikov et al	2014	Myocardial infarction and brain stroke	Russia	Barometric pressure	Yes
Plavcová and Kyselý,	2014	Cardiovascular disease	Czech Republic	Barometric pressure	Yes
Palmisano et al	2014	Bradyarrhythmia	Italy	Temperature	Yes
Ng et al	2014	Heatstroke	Japan	Temperature	Yes
McWilliams et al	2014	Mania and depression	Ireland	Wind barometric pressure, rainfall, temperature, and sunshine hours	No
Li et al	2014	Respiratory infection	China	Temperature	Yes
Chen et al	2014	Hand, foot, and mouth disease	China	Relative humidity, temperature, and rainfall	Yes
Flight et al	2014	Viral respiratory infection	England	Temperature and relative humidity	Yes
Wang and Lin	2015	Respiratory diseases, asthma, and chronic airway obstruction	Taiwan	Temperature	Yes
Vencloviene et al	2017	Acute coronary syndrome	Lithuania	Temperature, barometric pressure, relative humidity, and wind	Yes
Phung et al	2015	Diarrhoea	Vietnam	Relative humidity, temperature, and rainfall	Yes
Ozeki et al	2015	Headaches	Japan	Barometric pressure, temperature, relative humidity, and rainfall	Yes
Onozuka and Hagihara	2015b	Tuberculosis	Japan	Temperature	Yes
Onozuka and Hagihara	2015a	Influenza	Japan	Rainfall and relative humidity	Yes
Makra et al	2015	Asthma	Hungary	Temperature and humidity	Yes
Lim et al	2015	Dehydration	Korea	Temperature	Yes
Li et al	2015	Lung infection	China	Temperature	Yes
Khalid and Ghaffar	2015	Dengue	Pakistan	Rainfall, temperature, and wind	Yes
Condemi et al	2015	Renal colic and urinary calculi	Italy	Temperature	Yes
Çevik et al	2015	Stroke	Turkey	Temperature	Yes
Akpinar-Elci et al	2013	Asthma	Caribbean	Rainfall and wind	Yes
Yang et al	2016	Renal colic and urinary calculi	China	Temperature	Yes
Taheri Shahraiyni et al	2016	Acute aortic dissection	Berlin	Temperature and cloud cover	Yes
Royé et al	2016	Respiratory disease	Spain	Temperature, rainfall, and relative humidity	Yes
Hervás et al	2016	Streptococcal pharyngitis	Spain	Temperature, relative humidity, rainfall, atmospheric pressure, and wind	Yes
Gao et al	2016	Hepatitis A virus	China	Rainfall	Yes
Duan et al	2016	Scarlet fever	China	Temperature	Yes
Cheng et al	2016	Hand, foot, and mouth disease	China	Temperature	Yes
Zhao et al	2017	Hand, foot, and mouth disease	China	Relative humidity, barometric pressure, and rainfall	No
Wang et al	2017	Influenza	China and Canada	Relative humidity, temperature	Yes
Vencloviene et al	2017	Acute coronary syndrome	Lithuania	Temperature	Yes
Tamerius et al	2017	Influenza	Nicaragua	Temperature	No
Rowell et al	2017	Parkinson’s disease	Australia	Temperature	Yes
Peultier et al	2017	Knee osteoarthritis pain	France	Temperature, rainfall, sunshine hours, relative humidity, atmospheric pressure, and wind	Yes
Näyhä et al	2017	Cardiorespiratory disease	Finland	Temperature	Yes
Mu et al	2017	Chronic obstructive pulmonary disease	China	Relative humidity and temperature	Yes
Kim and Kim	2017	Cardiac arrhythmias	Korea	Temperature	Yes
He et al	2017	Allergic rhinitis	China	Temperature and relative humidity	Yes
Gou et al	2017	Hand, foot, and mouth disease	China	Temperature	Yes
Gestro et al	2017	Otitis media	Italy	Temperature, relative humidity, atmospheric pressure, and wind	Yes
Elcik et al	2017	Migraine headaches	USA	Wind	No
Čulić	2017	Cardiac arrhythmias	Croatia	Temperature	No
Azcárate and Mendoza	2017	Hypertension	Mexico	Temperature	Yes
Almendra et al	2017	Circulatory system diseases	Portugal	Temperature and wind	No
Acquaotta et al	2017	Haemolytic-uraemic syndrome	Italy	Temperature, rainfall, and relative humidity	Yes
Abbas et al	2017	Crimean-congo hemorrhagic fever	Pakistan	Temperature	Yes
Davis and Enfield	2018	Influenza	USA	Temperature	Yes
Tapak et al	2018	Depressive disorder, bipolar, and schizophrenia	Iran	Rainfall, snowfall, relative humidity, and cloud cover	Yes
Russo et al	2018	Legionnaires’ disease	Portugal	Relative humidity and temperature	Yes
Liu et al	2018	Hand, foot, and mouth disease	China	Temperature	No
Li et al	2018	Influenza	China	Temperature	Yes
Lam et al	2018	Chronic obstructive pulmonary disease and pneumonia	China	Relative humidity and temperature	Yes
Goldie et al	2019	Cardiovascular disease, respiratory disease, and renal disease	Australia	Relative humidity, wind, and temperature	Yes
Ge et al	2018	Rheumatic heart disease	China	Weather temperature	Yes
Davis and Enfield	2018	Respiratory disease	USA	Relative humidity and temperature	Yes
Brandl et al	2018	Psychiatric disorders	Germany	Temperature	Yes
Acharya et al	2018	Dengue fever	Nepal	Temperature	Yes
Yuan et al	2019	Dengue	Taiwan	Temperature and humidity	Yes
Xie et al	2019	Respiratory disease	China	Relative humidity	Yes
da Silva et al	2019	Asthma and bronchitis	Brazil	Temperature, barometric pressure, relative, and humidity	Yes
Romaszko et al	2019	Respiratory infection	Poland	Temperature	Yes
Liu et al	2019	Influenza	China	Relative humidity	Yes
Cui et al	2019	Cardiovascular disease	China	Temperature	Yes
Zhao et al	2020	Chronic pharyngitis	China	Relative humidity	Yes
Zhang et al	2020	Pertussis infection	China	Temperature and rainfall	Yes
Xie et al	2020	Rheumatoid arthritis	China	Rainfall	Yes
Wang et al	2020	Allergic rhinitis	China	Temperature and humidity	Yes
Vencloviene et al	2020	Acute myocardial infarction	Lithuania	Wind	Yes
Oh et al	2020	Benign paroxysmal positional vertigo	Korea	Humidity, temperature, atmospheric pressure, cloud cover, and sunshine	Yes
Nguyen et al	2020	Hand, foot, and mouth disease	Vietnam	Temperature, humidity, and rainfall	Yes
Matthew	2020	Malaria	Nigeria	Rainfall	Yes
Madaniyazi et al	2020	Cholesterol	China	Temperature	Yes
Hossain et al	2020	Pneumonia	Bangladesh	Relative humidity and temperature	No
Chang et al	2020	Asthma	China	Relative humidity	Yes
Chai et al	2020	Respiratory disease	China	Temperature	Yes
Bal and Sodoudi	2020	Dengue	India	Temperature	Yes
Xin et al	2021	Dysentery	China	Rainfall	Yes
Wang et al	2021	Bacillary dysentery	China	Temperature	Yes
Silva et al	2021	Respiratory disease	Portugal	Wind	Yes
Riancho et al	2021	Neurodegenerative diseases (Alzheimer disease, Parkinson’s disease, and amyotrophic lateral sclerosis)	Spain	Temperature	No
Nili et al	2021	Cutaneous leishmaniasis	Iran	Precipitation, temperature, and relative humidity	No
Ngo et al	2021	Acute lower respiratory infection	Vietnam	Temperature	Yes
Molina-Gómez et al	2021	Respiratory disease	Bogotá	Relative humidity	Yes
Meng et al	2021	Dengue	China	Rainfall	Yes
Martinaitiene and Raskauskiene	2021	Coronary artery disease	Lithuania	Temperature	Yes
Lindner-Cendrowska and Bröde	2021	Influenza	Poland	Temperature air pollutants	Yes
Lei et al	2021	Asthma	China	Temperature	Yes
Jahan et al	2021	Schizophrenia	Australia	Temperature, rainfall, and relative humidity	No
Gutierrez	2021	Leptospirosis	Colombia	Temperature and rainfall	Yes
Fdez-Arróyabe et al	2021	Influenza	Spain	Circulation weather	No
Dong et al	2021	Respiratory disease	China	Wind	Yes
Cheng et al	2021	Dengue	China	Temperature, rainfall, and relative humidity	Yes
Chaturrvedi and Dwivedi	2021	Malaria	India	Temperature	Yes
Huang et al	2022	Rheumatoid arthritis	China	Temperature	Yes
Ma et al	2022	Influenza A and B	China	Temperature, relative humidity, and wind	Yes
Vaičiulis et al	2022	Stroke	Lithuania	Temperature	Yes

Over the first three decades of the journal, only 21 publications (11.7%, *n* = 180) focused on a specific disease in the context of a meteorological variable (Fig. 2). As is the case for all papers on climate and health generally, from the early 2000s there has been a considerable increase in studies that use statistical methodologies to assess the relationships between climate and human health, aligning with the findings of McGregor (2012), with 18 published in 2017 alone (Fig. 2). Before this, the highest peak for 1 year at five papers (2.8%, *n* = 180) was for 1988 (Fig. 2). These outcomes are ascribed to the journal’s overall rise in articles as a result of its rise in the number of issues per volume. The increase in health and climate publications in the journal may potentially also be in response to disease outbreaks. Following 1988, there was the COVID-19 pandemic from 2020,the Zika virus epidemic in South America in 2015, and the Ebola outbreak in central Africa in 2015. There are 39 papers on COVID-19 and the Zika virus in the journal that were published after 2015. Health and climate publications substantially increased during this time.

## Geographical distribution of the research regions

**Fig. 3:**
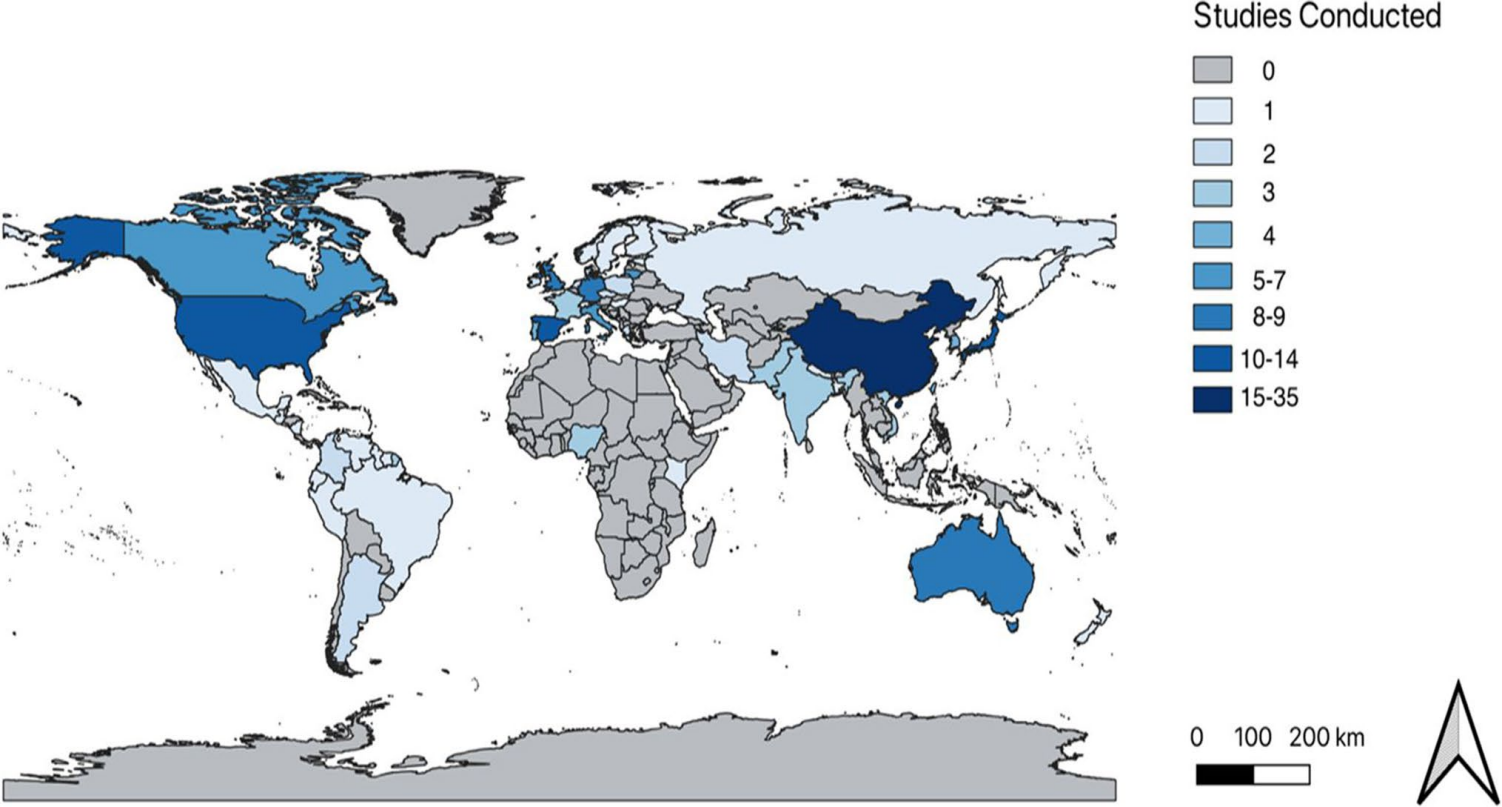
Countries of interest in health biometeorological studies

The 180 papers empirically studying climate and health since the inception of *The International Journal of Biometeorology* in 1957 to April 2022 are largely concentrated across much of the Northern Hemisphere (Fig. 3). The greatest proportion of the studies have been conducted in Asia (42.2%, *n* = 180). On the scale of individual countries, the greatest number of studies have been conducted in China (19.4%, *n* = 180), followed by Japan (8.5%, *n* = 180). In 2008, the CHC held a meeting in Japan, and in 2019 the *International Journal of Biometeorology* dedicated a special issue to Asian Biometeorology (Fdez-Arroyabe and Robau 2017), both of which may contribute to the large focus on Asia. These publications focused on a range of diseases. Some included chronic and acute respiratory diseases (24%, *n* = 75), genetic disorders (2.6%, *n* = 75), and vector-borne diseases (9%, *n* = 75) (Table 1).

The second largest continental representation in the published research is in Europe (35.6%). This may be because researchers were predominantly based in Europe before the ISB expanded to other parts of the world. Studies conducted in Europe have been concentrated in Spain (6.1%, *n* = 180) and Lithuania (3.3%, *n* = 180). European studies particularly dominated the earlier volumes of the *International Journal of Biometeorology* (Table 1). This was perhaps as Tromp was from Europe and worked predominantly in this field of research. In 2021, a special issue was dedicated to the first European Biometeorologists’ meeting, following which five papers on climate and health in the region were published. For the same year, however, a greater number of studies were conducted in North America (25), namely the USA (18) and Canada (7). Overall, papers from North America account for 13.3% (*n* = 180) of papers on climate and health in the *International Journal of Biometeorology.*

A small proportion of the literature on health biometeorology in the journal originates from the Southern Hemisphere. Only 10 studies have been conducted in Australia, and four in Africa. The latter comprises three studies in Nigeria and one in Kenya, leaving 52 African countries that have not been studied. Studies conducted in Africa compromise 4.1% (*n* = 180) of all papers in the journal in total. However, whilst this fits proportionately with the journal figures (Fitchett 2021), it is surprising given the heightened disease load in Africa, particularly to climate-sensitive conditions such as malaria, Ebola, and yellow fever (Sen Roy 2018; de Villiers 2021).

Studies based in South America compromise 8.3% of the total 180 papers (Fig. 3). It is interesting to note that although there was a special issue on Latin America/Caribbean Biometeorology in 2018 and a special issue on the Brazilian Congress in 2019, very few of the publications from either special issue explored health biometeorology of specific diseases (Fdez-Arroyabe and Robau 2017).

## Diseases under investigation

**Fig. 4:**
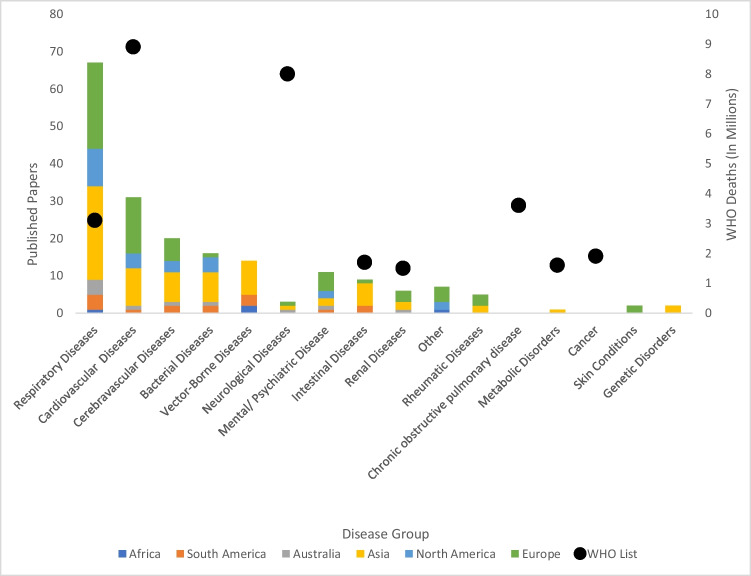
Diseases studied per continent and WHO’s top ten list of leading causes of death globally

Across the 180 papers (1957–2022), a total of 14 disease groups were explored: neurological, cardiovascular, skin, rheumatic, intestinal, metabolic, psychiatric, vector-borne, genetic, cerebrovascular, respiratory, renal, and bacterial diseases and others such as measles (Fig. 4). According to WHO (2020) respiratory diseases are the fourth-ranked leading cause of death notably respiratory diseases represented the greatest proportion studied (37.2%, *n* = 180), with research on all six of the habitable continents, including both chronic respiratory conditions such as asthma and acute respiratory conditions such as influenza. As there has been a lot of research conducted on respiratory diseases and it is well-understood that climate and weather influence their transmission dynamics. These studies, similar to the overall trends, were concentrated in the Northern Hemisphere, with 25 papers on respiratory health from Asia and 23 from Europe (Fig. 4). The dominance of research from Asia in this domain is accounted for, in part, by the SARS and MERS epidemics in the region (Reichert et al. 2004). According to the WHO top 10 causes of death, stroke (a neurological disease) is the second leading cause of death globally; however, only 5% (*n* = 180) of the studies published in the International Journal of Biometeorology examined strokes.

Cardiovascular diseases are the leading cause of death according to the WHO (2020) and were responsible for 8.9 million deaths globally in 2019. However, studies on cardiovascular diseases such as myocardial infarctions only represent the second most frequently explored group (17.2%, *n* = 180). This is likely as a result of an increase in research on non-communicable diseases in the twenty-first century and the fact that cardiovascular diseases are the major cause of mortality (Münzel et al. 2022). These were conducted predominantly in Europe (48.4%, *n* = 15). This is likely due to the ageing population of the region, heightening the vulnerability to these diseases (Murphy 2017).

Studies conducted in South America predominantly explored vector-borne diseases such as dengue and Malaria and intestinal diseases such as diarrhoea which are ranked eighth on the WHO top 10 causes of death lost, due to both socioeconomic and geographical factors (WHO 2020; Ruano et al. 2021). By contrast, whilst most of the disease categories were investigated in Europe, vector-borne diseases were not, again a result of the climatic and geographical factors determining disease prevalence (Roclöv and Dubriw 2020). The four studies for Africa explored measles, malaria, and asthma.

Psychiatric conditions, which represent 6.1% of the total 180 papers, are concentrated in the European region, with none from Africa or South America (Fig. 4). All disease groups were explored in at least one Asian country except for dermatological conditions, which were only investigated in Europe, since the skin is the organ that is exposed to the environment the most (Balato et al. 2014; Richard et al. 2022). Although stroke and chronic obstructive pulmonary disease are the second and third leading causes of death respectively according to the WHO (2020) no studies published in the International Journal of Biometeorology examined chronic obstructive pulmonary disease, these publication trends are interesting as they do not coincide with the WHO top ten list of the leading causes of death globally (Fig. 4: WHO 2020). Cancer is ranked sixth on the list of leading causes of death; however, no study examined cancer.

This review also assessed if the 180 studies conducted respectively yielded any statistical significance between the variable under investigation in each paper. Of the tests for relationships between the disease and specific meteorological variables only 14.4% (*n* = 180) of the papers being investigated in this review report results that are not statistically significant (Table 1). This may reveal the bias in publication towards only those studies which reveal statistically significant relationships, in the expected direction, that have been recorded in the biometeorological research on phenology (Menzel et al. 2006). If this is the case, it would obscure results of repeated studies that may contest relationships between specific diseases and meteorological conditions, whether generally or for a specific region.

**Fig. 5:**
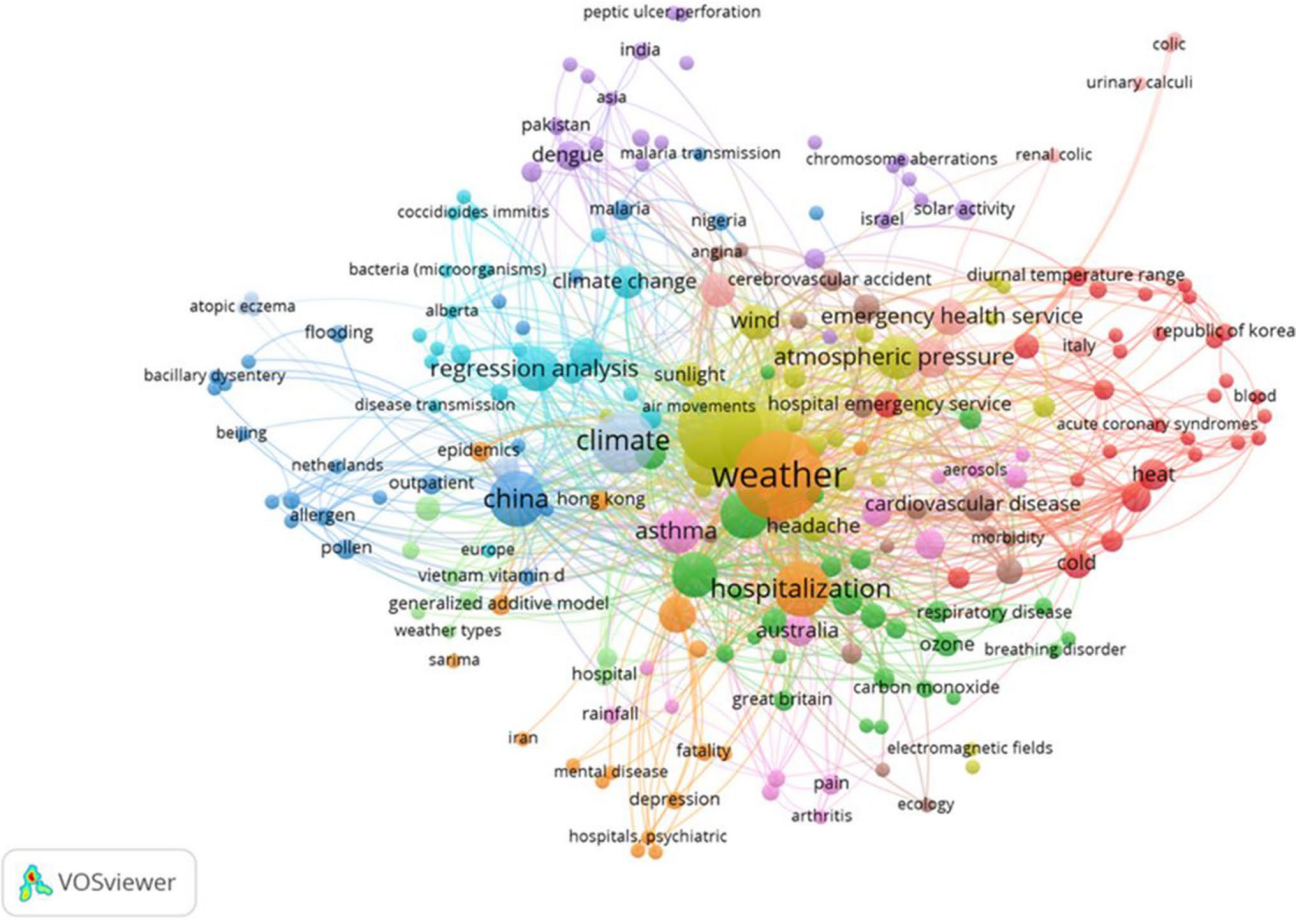
Bibliometric visualization map of all 180 papers

An increasing body of evidence exists regarding how climate change may affect human health, notably the emergence and spread of diseases. Understanding this connection is crucial because it will increase the already heavy burden that diseases place on the country's economy and public health. For instance, heat and acute coronary syndromes, climate change and malaria, solar activity, and chromosome aberrations (Fig. 5). These findings illustrate further that research outputs were situated greatly in China (Asia) and the weather was the dominant meteorological variable, and the majority of the papers utilized statistical methodologies in the form of regression analysis to determine the relationship between climate and health (Fig. 5). Researchers achieved this by utilizing hospitalization data (Fig. 5). Researchers examined the link between temperature and cardiovascular disease in nations like Italy and Korea, and those in the Middle East (Israel) evaluated the link between solar activity and chromosome abnormalities (Fig. 5). Overall diseases differ by demographic and geographic location, and they evolve with time.

**Fig. 6:**
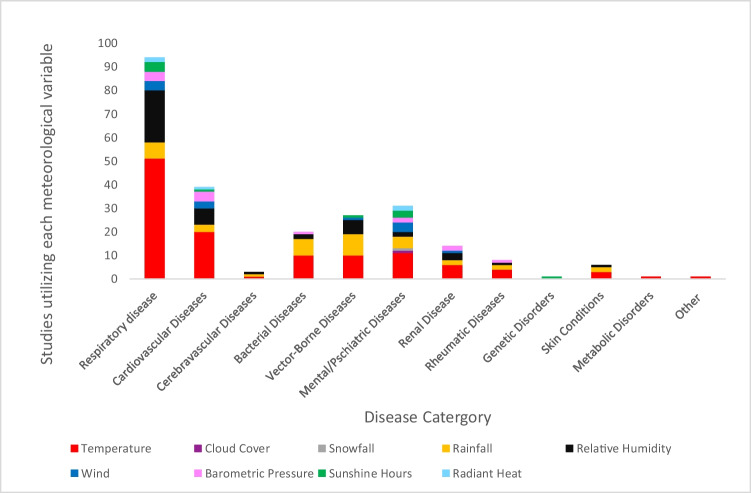
Meteorological variables studied per disease category

A range of meteorological variables is explored across the 180 papers spanning from the year 1957 to April 2022. In each of the disease types, average temperature, relative humidity, and rainfall were most commonly examined (Fig. 6). With the exception of genetic disorders, studies examining all disease categories used the meteorological variable temperature. Whilst only the category of mental/psychiatric diseases used cloud cover as a meteorological variable. Temperature, precipitation, and relative humidity variables were evaluated for both skin conditions and cerebrovascular diseases. All the meteorological variables were examined in studies on respiratory and cardiovascular diseases, but not snowfall. Studies investigating vector-borne diseases looked at the amount of sunshine, the relative humidity, the amount of rain, and the temperature. Temperature, rainfall, relative humidity, and barometric pressure were all evaluated in studies on bacterial and rheumatic diseases (Fig. 6).

## Key research directions in biometeorology of health

This review explores the publications that analysed the relationship between specific, diagnosable diseases, and meteorological variables. Since the inception of the journal, there has been an increase in publications that assess the relationship between meteorological variables and diagnosable diseases. This increase in research is imperative in the context of climate change and concerns about the climatic sensitivity of conditions such as COVID-19 (Smit et al. 2020). Warming and increases in humidity are projected to create new habitats for diseases like malaria in Africa (Epstein et al. 1998; Martens 1999), parasitic nematodes in the Arctic (Kutz et al. 2005), West Nile virus (Reisen et al. 2006), Lyme disease in North America (Ogden et al. 2008), and schistosomiasis in China. These projections are largely based on studies (Zhou et al. 2008). In the East African highlands, for instance, reported altitudinal increases in falciparum malaria over the past 30 years have been linked to rising temperatures and are compatible with theories of anopheline mosquito vector development (Pascual et al. 2006). This will be exacerbated by climate change's effects on the length of seasons.

This review has highlighted that research on climate and health in the *International Journal of Biometeorology* is highly concentrated in the Northern Hemisphere (Fig. 6), whilst many countries in the Southern Hemisphere carry a greater disease burden (Valodia and Taylor 2022), have an overburdened public health sector, and are projected to experience an above-average rate of climate warming in decades to come (IPCC 2022 6AR). The importance of health research in low- and middle-income nations has been highlighted by recent incidences of developing and reemerging infectious illnesses, commitments to obtaining universal health coverage, and increased interest in global health (Ranabhat et al. 2020). Additional factors to take into account in the context of evolving population health demands include the requirement for contextualized evidence to produce local solutions, innovation to increase efficiency, and the creation of more effective treatment regimens given the rise in drug resistance (Nabyonga-Orem et al. 2021). Contextual elements such as conflict settings (Bowsher et al. 2019), multilayered governance in implementation research (Patel et al. 2017), and rapidly developing digital technologies necessitate ongoing adaptation of health research systems to ensure relevance and effectiveness (Kostkova 2015). Since they are currently the least studied, these nations need the greatest attention both now and in the future. Health research is essential for advancing development, equity, and health (Nuyens 2005). Inadequate financing, infrastructure, and skill levels, disproportionate rates of climate change and lack of adaptation given its unique disease burden as well as poor governance of health research are the key causes of Africa’s subpar research capacity, which has long been a cause for concern (Fig. 5; Chu et al. 2014; Rusakaniko et al. 2019; Simpkin et al. 2019). This may help to explain why so little research is produced; Africa only contributes 2% (Schemm 2013) to global research output and 1.3% to worldwide publications (Uthman et al. 2015). Whilst this can be facilitated through regional conferences, special issues, and collaborative research (WHO 2018; Fitchett 2021), this will also need greater focus in funding for research in these regions.

It is also important that local researchers from African and South American countries be included and involved in this research. The increase in research from the Northern Hemisphere has resulted in the development of strong methodological approaches that can be replicated in studies in regions such as Africa (Sen Roy 2018; Marincola and Kariuki 2020; de Villiers 2021). Whilst it does increase the total research output it often misses nuances (Marincola and Kariuki 2020). Particularly in Africa and South America, key nuances in health related to the prevalence of endemic and regionally occurring diseases (Moreira et al. 2020), such as malaria, Ebola, yellow fever, Zika virus, and chikungunya virus (de Villiers 2021; Roclöv and Dubriw 2020; Ruano et al. 2021). It is therefore critical that collaboration is inclusive, and conducted on the ground, rather than perpetuating parachute science that lacks local context (Stefanoudis et al. 2021).

Whilst research on climate and health has been central to the focus of both the International Society of Biometeorology and the *International Journal of Biometeorology*, many other journals do publish research on these topics, including but not limited to *Health Communication, the International Journal of Environmental Research and Public Health, The Lancet, and Global Health Action*, in addition to local and international interdisciplinary journals. This review does not consider those papers, as the aim is to explicitly track trends within the journal. However, these key avenues for future research would be implemented across research published in this much wider range of journals. Important in further developing Biometeorology as a discipline that includes climate and health is raising awareness amongst researchers who are working in this nexus of the International Society of Biometeorology as a forum for engagement, and the *International Journal of Biometeorology* as an outlet for publication. As an international journal, it is important to strive towards greater and more balanced international representation in published research.

As a factor of the inclusion criteria, all 180 papers considered in this review employed statistical methodologies to quantify the impacts of climate on health (Table 1). This makes the studies more robust than earlier more qualitative assessments of this nexus, but often, it ignores the qualitative or experiential and aetiological aspects of these relationships (Mehta 2022). Firstly, purely statistical and empirical studies do not study the nature of the phenomenon itself; it does not engage with individual items thus making it difficult to infer causation and are limited to correlation only (Musani et al. 2007; Mehta 2022).

Secondly, a wide range of statistical methods is being employed across these studies (e.g., Poisson regression, times series analysis, linear regression, generalized additive models, and distributed-lag nonlinear model). This makes the comparison of results from studies difficult to perform. So, whilst statistical analyses of empirical case data are important, they do not negate the importance of laboratory work in understanding aetiology, and qualitative work on experiential components of seasonality. An important future direction, in this regard, is to employ a less reductive approach and to focus more holistically on the physiological reasons that underpin any relationships found and reported from the data. This would be most effectively achieved through greater collaboration between climatologists, healthcare practitioners, and data scientists. Indeed, this was one of the key aims of the society at its inception (Tout 1987; Sheridan and Allen 2017).

## Conclusion

A significant increase in human health papers has been published in the last decade in the *International Journal of Biometeorology*. Over the full period of publication of the journal, studies dedicated to the intersection between specific, diagnosable diseases and meteorological variables account for only 4.1% of the 4350 works of the journal to date. Although the criteria for this review were specific, the 180 papers analysed are an indication that the International Journal of Biometeorology has indeed stuck to its scope in documenting such studies. Researchers in Europe and Asia have done an excellent job in documenting their studies in the journal since its inception, contributing a combined 77.8% to the total 180 studies. It is important to have more studies explore the extent to which diseases are climate sensitive. Of the 180 papers reviewed, only four (2.22%) studies were based in Africa. A total of 15 (8.3%) studies were based in South America. This means that regionally endemic conditions such as Ebola, yellow fever, and malaria are largely unstudied in this journal. Given a change in climatic conditions, continents such as South America and Africa, which are affected greatly by climate change have minimal to no studies on climate and health. In Africa and South America, dysfunctional health systems and the collision of epidemics of communicable and non-communicable diseases have exacerbated this. The paucity of research on human biometeorology conducted in Africa and South America was exposed by this systematic literature review, which also identified shortcomings that may serve as the basis for future studies. The study had the advantage of making it clear which sectors should receive the majority of the funding for research in the near future. In this way, gaps in publication production can promote the expansion of research in specific subregions of South America and Africa.

## Supplementary Information

Below is the link to the electronic supplementary material.

Supplementary file1 (DOCX 46 KB)
