# Supplementary material for: Climate and human health: a review of publication trends in the International Journal of Biometeorology
Source: Int J Biometeorol. 2023 May 2;67(6):933–55. doi: 10.1007/s00484-023-02466-8 (PMC10153057; doi:10.1007/s00484-023-02466-8)
Supplement: Supplementary file 1 — Supplementary file1 (DOCX 46 KB) [file 484_2023_2466_MOESM1_ESM.docx]

Supplementary Table 1: Publications on the intersection of specific diagnosable diseases and meteorological variables in the International Journal of Biometeorology under review.

| Author | Year | Disease | Country | Meteorological Variable | Statistical significance |
| --- | --- | --- | --- | --- | --- |
| Borreguero | 1957 | Allergic Diseases | Spain | Weather | Yes |
| Tromp | 1957 | Bronchial Asthma and Mental Diseases | Netherlands | Weather | Yes |
| Whiten | 1957 | Rheumatism | Great Britain | Weather | Yes |
| Derrick | 1965 | Asthma | Australia | Weather | Yes |
| Tromp and Bouma | 1966 | Arthritic Pain | Netherlands | Weather | Yes |
| Paulus and Smith | 1967 | Allergic Bronchial Asthma | Minnesota | Weather and Air Pollutants | Yes |
| Hansen and Pedersen | 1972 | Perforated Duodenal Ulcer | Denmark | Barometric Pressure | No |
| Gomersall and Stuart | 1973 | Migraines | Scotland | Weather | Yes |
| Bodhe and Mokashi | 1975 | Peptic Ulcer | India | Weather (Vapour Pressure) | Yes |
| Specht et al | 1975 | Asthma | Australia | Botanical Causes | No |
| Von Deschwanden and Jungmann | 1975 | Mental Illness (Schizophrenia and Depression); Hay Fever; Asthma | Germany; London; Utah | Cold and Warm Fronts; Temperature; Temperature and Ambient Air Pollution | Yes |
| Shiffman et al | 1976 | Intestinal Disease (Diarrhoea) | Guatemala | Rainfall and Temperature | Yes |
| Fleischer and Asnani | 1978 | Asthma | Nairobi | Rainfall and Temperature | Yes |
| Schulman et al | 1980 | Headaches | Boston | Barometric Pressure | No |
| Osterman et al | 1981 | Headaches | Sweden | Weather | Yes |
| Goldstein | 1981 | Asthma | New York City and New Orleans | Weather | Yes |
| Deacon and Williams | 1982 | Sudden Infant Death Syndrome | 8 Metropolitan Communities | Weather (Precipitation and Temperature) | Yes |
| Formiconi and Tagliaferri | 1984 | Urinary Stone Colic | Florence, Italy | Weather (Temperature, Precipitation and Barometric Pressure) | Yes |
| Mukammal et al | 1984 | Cardiovascular Disease (Ischaemic Heart Disease) | Toronto, Canada | Thermal Load Index | Yes |
| Bellossi et al | 1985 | Myocardial Infarction | France | Geomagnetic Activity | No |
| Fujita | 1987 | Urinary Stone Colic | Tokyo | Weather (Temperature and Barometric Pressure) | Yes |
| Fletcher | 1988 | Föhn  Illness | Canada | Weather (Temperature and Wind Velocity) | Yes |
| Michael et al | 1988 | Depression | Minnesota and California | Weather (Winter Temperature) | No |
| Mannino and Washburn | 1988 | Cardiovascular Disease | Wisconsin | Weather (Temperature and Precipitation) | Yes |
| Morton | 1988 | Headaches | Canada | Earthquakes (Air Ions) | Yes |
| Suzuki et al | 1988 | Asthma | Japan | Weather (Temperature and Barometric Pressure) | Yes |
| Collier | 1992 | Meningococcal Meningitis | United Kingdom | Weather (Temperature and Air Quality) | Yes |
| Ohtsuka et al | 1995 | Diabetes Mellitus | Japan | Weather (Temperature) | Yes |
| Halpern et al | 1995 | Foetal Chromosome abnormalities (Trisomy - 21) | Tel Aviv, Israel | Solar Activity | No |
| Thompson et al | 1996 | Myocardial Infarction | Leicester, England | Weather (Temperature and Humidity) | Yes |
| Aikman | 1997 | Arthritis (Rheumatoid and Osteoarthritis) | Bendigo, Australia | Weather (Temperature, Precipitation and Barometric Pressure, Relative Humidity) | Yes |
| McGregor et al | 1999 | Respiratory Disease | Birmingham, United Kingdom | Weather ( Winter Air Mass) | Yes |
| Rutherford et al | 1999 | Asthma | Brisbane, Australia | Wind (Dust Particles) | Yes |
| Bentham and Langford | 2001 | Food Poisoning | England and Wales | Weather (Temperature) | Yes |
| Laaidi | 2001 | Pollinosis | Burgundy, France | Weather (Wind, Humidity, Precipitation and Temperature) | Yes |
| Schreiber | 2001 | Dengue | San Juan, Puerto Rico | Weather (Temperature and Humidity) | Yes |
| Vaitl | 2001 | Headaches and Migraines | Giessen, Germany | Sferics | Yes |
| Vocks et al | 2001 | Atopic Eczema | Davos, Switzerland | Weather (Temperature) | Yes |
| Gagnon et al | 2002 | Malaria | Colombia, Ecuador, French Guiana, Guyana, Peru, Suriname and Venezuela | El Nino Southern Oscillation | Yes |
| Makie et al | 2002 | Cerebrovascular Disease, Respiratory Disease, Digestive Diseases | Fukuoka, Japan | Weather (Temperature and Barometric Pressure) | Yes |
| Rusticucci et al | 2002 | Cerebrovascular Disease, Respiratory Disease, Digestive Diseases, Muscle Pains, Skin and Allergies, Neurological Diseases and Psychopathological Disorders | Buenos Aires, Argentina | Weather (Temperature and Precipitation) | Yes |
| Schlink et al | 2002 | Respiratory Disease | Leipzig, Germany | Weather (Global Radiation, Temperature, Vapour Pressure, Air Humidity) | Yes |
| Wang et al | 2002 | Stroke | Toyama Prefecture, Japan | Seasons | No |
| Kolivras and Comrie | 2003 | Coccidioidomycosis (Valley Fever) | Arizona, USA | Weather (Temperature and Precipitation) | Yes |
| Tobías | 2003 | Asthma | Madrid, Spain | Photochemical air pollutants | No |
| Ebi et al | 2004 | Cardiovascular Diseases (Myocardial Infarction, Angina Pectoris, Congestive Heart Failure) and Stroke | Los Angeles, San Francisco and Sacramento, California | El Nino Southern Oscillation, Temperature and Precipitation | Yes |
| Ohwaki et al | 2004 | Hypertensive Intracerebral Haemorrhage | Japan | Weather | No |
| Bulbena et al | 2005 | Anxiety | Barcelona, Spain | Weather (Wind Speed, Wind Direction, Precipitation, Temperature, Humidity and Solar Radiation | Yes |
| Gyan et al | 2005 | Asthma | Trinidad | African Dust Clouds | Yes |
| Kovats et al | 2005 | Campylobacter | Canada, Scotland, Australia, Malta, Denmark, Spain, Czech Republic, Estonia, Greece, Ireland | Weather (Temperature) | Yes |
| Stoupel et al | 2005 | Down Syndrome | Israel | Cosmic Ray Activity | Yes |
| Villeneuve et al | 2005 | Asthma | Ottawa, Canada | Weather (Relative Humidity, Temperature, Precipitation) | Yes |
| Fleury et al | 2006 | Bacterial Enteric Infection | Canada | Weather (Temperature) | Yes |
| Hrushesky et al | 2006 | Uterine Cervical Human Papilloma Virus | Holland | Seasons | Yes |
| Nastos and Matzarakis | 2006 | Respiratory Infection | Athens, Greece | Weather (Temperature, Relative Humidity, Absolute Humidity, Sunshine, Wind Speed and Barometric Pressure) | Yes |
| Ohshige et al | 2006 | Stroke | Yokohama, Japan | Weather (Temperature, Humidity and Barometric Pressure) | Yes |
| Zender and Talamantes | 2006 | Coccidioidomycosis (Valley Fever) | Kern, California | Weather (Precipitation and Temperature) | Yes |
| Liang et al | 2007 | Acute Coronary Syndrome | Taiwan | Weather (Temperature) | Yes |
| Nakaguchi et al | 2008 | Intracerebral Haemorrhage | Shin-ichi Town, Japan | Weather (Barometric Pressure, Temperature, Humidity, Precipitation, Wind velocity and Wind direction) | Yes |
| Prospero et al | 2008 | Asthma | Caribbean | Atlantic Trade Winds and African Dust | No |
| Suárez-Varela  et al | 2008 | Atopic Eczema | Spain | Weather (Precipitation, Temperature, Precipitation, Relative Humidity) | Yes |
| García-Marcos  et al | 2009 | Asthma | Spain | Weather (Relative Humidity, Temperature) | Yes |
| Liang et al | 2009 | Chronic Obstructive Pulmonary Disease | Taiwan | Weather (Temperature) | Yes |
| Styra et al | 2009 | Cardiovascular Disease | Vinius City, Lithuania | Cosmic Ray Flux and Atmospheric Pressure | No |
| Wang et al | 2009 | Stroke | Brisbane, Australia | Weather (Temperature) | Yes |
| Arnedo-Pena | 2011 | Asthma and Allergies | Spain | Sunny Hours | Yes |
| Azevedo et al | 2011 | Respiratory and Cardiovascular diseases | Portugal | Ozone Trajectories | Yes |
| Omonijo et al | 2012 | Measles | Ondo State, Nigeria | Human-Biometeorological Parameters (Thermal Environment) | Yes |
| Goggins et al | 2012 | Stroke | Hong Kong, China | Weather (Temperature, Humidity, Solar Radiation, Precipitation, Air Pressure) | Yes |
| Ferrari et al | 2012 | Obstructive Pulmonary Disease | Bavaria, Germany | Weather (Air Pressure, Humidity, Solar Radiation, Temperature and Wind Speed) | Yes |
| Coelho and Massad | 2012 | Leptospirosis | São Paulo  , Brazil | Weather (Temperature and Precipitation) | Yes |
| Alexander | 2013 | Heart disease, Arrhythmia, Heart Failure, Cardiopulmonary Arrest, Angina, Pectoris, Psychiatric Diseases, Stroke, Transient Ischemic Attack | Buenos Aires, Argentina | Weather and Seasonality | Yes |
| McWilliams et al | 2013 | Psychotic Illnesses | Ireland | Weather (Wind Speed, Wind Direction, Precipitation, Temperature, Humidity, Solar Radiation and Barometric Pressure) | No |
| Scheidt et al | 2013 | Migraines | Germany | Weather (Temperature) | Yes |
| Bakal et al | 2013 | Acute Coronary Syndrome | Global | Weather (Temperature and Humidity) | Yes |
| Arnedo-Pena | 2013 | Asthma | Western Europe | Weather (Temperature, Precipitation, Sunshine and Humidity) | Yes |
| Lim et al | 2013 | Cardiovascular Disease | Seoul, Korea | Weather (Temperature) | Yes |
| Soyiri et al | 2013 | Asthma | London | Weather (Temperature, Ozone and Air Quality) | No |
| Akinbobola and Omotosho | 2013 | Malaria | North Central Nigeria | Weather (Temperature, Rainfall and Relative Humidity) | Yes |
| Yackerson et al | 2014 | Schizophrenia | Israel | Air- suspended particulate concentration | Yes |
| de Weger et al | 2014 | Allergic Rhinitis | Netherlands | Weather (Temperature) | Yes |
| Wanka et al | 2014 | Respiratory Disease | Munich, Germany | Meteorological Variables | No |
| Smedslund et al | 2014 | Fibromyalgia Pain | Norway | Weather (Barometric Pressure, Temperature, Relative Humidity and Solar Flux) | Yes |
| Shaposhnikov et al | 2014 | Myocardial Infarction and Brain Stroke | Moscow, Russia | Barometric Pressure and Geomagnetic Factors | Yes |
| Plavcová and Kyselý, | 2014 | Cardiovascular Disease | Prague, Czech Republic | Air Pressure | Yes |
| Palmisano et al | 2014 | Bradyarrhythmia | Lecce, Italy | Seasons and Temperature | Yes |
| Ng et al | 2014 | Heatstroke | Kanto, Japan | Summer Temperature | Yes |
| McWilliams et al | 2014 | Mania and Depression | Dublin, Ireland | Weather (Wind Speed, Wind Direction, Barometric Pressure, Rainfall, Temperature and Hours of Sunshine) | No |
| Li et al | 2014 | Respiratory Infection | Guangzhou, China | Weather (Diurnal Temperature Range) | Yes |
| Chen et al | 2014 | Hand, Foot and Mouth Disease | Guangzhou, China | Weather ( Relative Humidity, Temperature, Precipitation) | Yes |
| Flight et al | 2014 | Viral Respiratory Infection | Manchester, UK | Weather (Temperature and Humidity) | Yes |
| Wang and Lin | 2015 | Respiratory Diseases, Asthma and Chronic Airway Obstruction | Taiwan | Weather (Temperature) | Yes |
| Vencloviene et al | 2015 | Acute Coronary Syndrome | Kaunas, Lithuania | Weather (Temperature, Barometric Pressure, Relative Humidity and Wind Speed) | Yes |
| Phung et al | 2015 | Diarrhoea | Mekong Delta, Vietnam | Weather (Relative Humidity, Temperature, Precipitation) | Yes |
| Ozeki et al | 2015 | Headaches | Shizuoka, Japan | Weather (Barometric Pressure, Temperature, Humidity and Precipitation) | Yes |
| Onozuka and Hagihara | 2015 | Tuberculosis | Japan | Weather (Temperature) | Yes |
| Onozuka and Hagihara | 2015 | Influenza | Fukuoka, Japan | Indian Ocean Dipole and El Nino Southern Oscillation | Yes |
| Makra et al | 2015 | Asthma | Szeged, Hungary | Weather (Temperature and Humidity) | Yes |
| Lim et al | 2015 | Dehydration | Seoul, Korea | Weather (Temperature) | Yes |
| Li et al | 2015 | Lung Infection | Baotou, China | Weather (Temperature) | Yes |
| Khalid and Ghaffar | 2015 | Dengue | Pakistan | Weather (Rainfall, Temperature and Wind Speed) | Yes |
| Condemi et al | 2015 | Renal Colic and Urinary Calculi | Cuneo, Italy | Weather (Temperature) | Yes |
| Çevik et al | 2015 | Stroke | Turkey | Weather (Temperature) | Yes |
| Akpinar-Elci et al | 2015 | Asthma | Grenada, Caribbean | Weather (Rainfall and Dust Particles) | Yes |
| Yang et al | 2016 | Renal Colic and Urinary Calculi | Guangzhou, China | Weather (Temperature) | Yes |
| Taheri Shahraiyni et al | 2016 | Acute Aortic Dissection | Berlin | Weather (Temperature and Cloud Cover) | Yes |
| Royé et al | 2016 | Respiratory Disease | Galicia, Spain | Weather (Temperature, Precipitation and Humidity) | Yes |
| Hervás  et al | 2016 | Streptococcal Pharyngitis | Mallorca, Spain | Weather (Temperature, Humidity, Rainfall, Atmospheric Pressure, Wind Speed and Solar Radiation) | Yes |
| Gao et al | 2016 | Hepatitis A Virus | Anhui Province, China | Weather (Precipitation - Floods) | Yes |
| Duan et al | 2016 | Scarlet Fever | Hefei City, China | Weather (Temperature) | Yes |
| Cheng et al | 2016 | Hand, Foot and Mouth Disease | Rural Hefei City, China | Weather (Temperature) | Yes |
| Zhao et al | 2017 | Hand, Foot and Mouth Disease | Huainan City, China | Weather (Relative Humidity, Barometric Pressure and Rainfall) | No |
| Wang et al | 2017 | Influenza | Shanghai, Hong Kong - China and British Columbia ( Canada) | Weather ( Relative Humidity, Temperature) | Yes |
| Vencloviene et al | 2017 | Acute Coronary Syndrome | Kaunas, Lithuania | Space Weather Conditions | Yes |
| Tamerius et al | 2017 | Influenza | Nicaragua | Indoor Temperature | No |
| Rowell et al | 2017 | Parkinson's Disease | Australia | Seasonal Temperature | Yes |
| Peultier et al | 2017 | Knee Osteoarthritis Pain | France | Weather (Temperature, Precipitation, Sunshine, Humidity, Atmospheric Pressure and Wind Speed) | Yes |
| Näyhä et al | 2017 | Cardiorespiratory Disease | Finland | Weather (Temperature) | Yes |
| Mu et al | 2017 | Chronic Obstructive Pulmonary Disease | Shanghai, China | Weather (Relative Humidity, Temperature) | Yes |
| Kim and Kim | 2017 | Cardiac Arrhythmias | Seoul, Korea | Weather (Temperature) | Yes |
| He et al | 2017 | Allergic Rhinitis | Shanghai, China | Weather (Temperature and Humidity) | Yes |
| Gou et al | 2017 | Hand, Foot and Mouth Disease | Gansu, China | Weather (Temperature) | Yes |
| Gestro et al | 2017 | Otitis Media | Italy | Weather (Temperature, Humidity, Atmospheric Pressure and Wind) | Yes |
| Elcik et al | 2017 | Migraine Headaches | North Carolina | Weather (Air Mass) | No |
| Čulić | 2017 | Cardiac Arrhythmias | Split, Croatia | Weather (Temperature) | No |
| Azcárate and Mendoza | 2017 | Hypertension | Mexico | Geomagnetic Activity | Yes |
| Almendra et al | 2017 | Circulatory System Diseases | Lisbon, Portugal | Winter North Atlantic Oscillation | No |
| Acquaotta et al | 2017 | Haemolytic-uraemic syndrome | Italy | Weather (Temperature, Rainfall and Humidity) | Yes |
| Abbas et al | 2017 | Crimean-Congo hemorrhagic fever | Pakistan | Weather (Temperature) | Yes |
| Davis and Enfield | 2018 | Influenza | USA | Weather (Temperature) | Yes |
| Tapak et al | 2018 | Depressive Disorder, Bipolar and Schizophrenia | Iran | Weather (Dust, Rain, Snow, Fog and Cloud Cover) | Yes |
| Russo et al | 2018 | Legionnaires' Disease | Portugal | Weather (Relative Humidity and Temperature) | Yes |
| Liu et al | 2018 | Hand, Foot and Mouth Disease | Nanjing, China | Weather (Temperature) | No |
| Li et al | 2018 | Influenza | Hong Kong, China | Weather (Temperature) | Yes |
| Lam et al | 2018 | Chronic Obstructive Pulmonary Disease and Pneumonia | Hong Kong, China | Weather (Relative Humidity and Temperature) | Yes |
| Goldie et al | 2018 | Cardiovascular Disease, Respiratory Disease and Renal Disease | Australia | Weather (Humidity, Dewpoint, Wind Speed and Temperature) | Yes |
| Ge at al | 2018 | Rheumatic Heart Disease | Shanghai, China | Weather (Temperature) | Yes |
| Davis and Enfield | 2018 | Respiratory Disease | Charlottesville, Virginia | Weather (Relative Humidity and Temperature) | Yes |
| Brandl et al | 2018 | Psychiatric Disorders | Berlin, Germany | Weather (Temperature) | Yes |
| Acharya et al | 2018 | Dengue Fever | Nepal | Land Surface Temperature | Yes |
| Yuan et al | 2019 | Dengue | Taiwan | Weather (Temperature and Humidity) | Yes |
| Xie et al | 2019 | Respiratory Disease | Hefei City, China | Air Pollutants | Yes |
| da Silva et al | 2019 | Asthma and Bronchitis | Canoas City, Brazil | Weather (Temperature, Barometric Pressure, Relative and Humidity) | Yes |
| Romaszko et al | 2019 | Respiratory Infection | Poland | Universal Thermal Climate Index (UTCI) | Yes |
| Liu et al | 2019 | Influenza | Hefei City, China | Air Pollutants | Yes |
| Cui et al | 2019 | Cardiovascular Disease | Hefei City, China | Weather (Temperature) | Yes |
| Zhao et al | 2020 | Chronic Pharyngitis | Xinxiang, China | Ambient Air Pollution | Yes |
| Zhang et al | 2020 | Pertussis Infection | Jinan City, China | Weather (Temperature and Rainfall) | Yes |
| Xie et al | 2020 | Rheumatoid Arthritis | Hefei City, China | Weather (Rainfall) | Yes |
| Wang et al | 2020 | Allergic Rhinitis | Beijing, China | Weather (Temperature and Humidity) | Yes |
| Vencloviene et al | 2020 | Acute Myocardial Infarction | Kaunas, Lithuania | Quasi-biennial Oscillation Phase, Solar Wind and Geomagnetic Activity | Yes |
| Oh et al | 2020 | Benign Paroxysmal Positional Vertigo | Seoul, Korea | Weather (Humidity, Temperature, Atmospheric Pressure, Cloud Cover and Sunshine) | Yes |
| Nguyen et al | 2020 | Hand, Foot and Mouth Disease | Delta Region, Vietnam | Weather (Temperature, Humidity and Rainfall) | Yes |
| Matthew | 2020 | Malaria | Nigeria | Weather (Rainfall) | Yes |
| Madaniyazi et al | 2020 | Cholesterol | Kailuan, China | Weather (Temperature) | Yes |
| Hossain et al | 2020 | Pneumonia | Bangladesh | Weather (Relative Humidity and Temperature) | No |
| Chang et al | 2020 | Asthma | Shenyang, China | Air Pollutants | Yes |
| Chai et al | 2020 | Respiratory Disease | Lanzhou, China | Weather (Temperature) | Yes |
| Bal and Sodoudi | 2020 | Dengue | Kolkata, India | Weather (Temperature) | Yes |
| Xin et al | 2021 | Dysentery | China | Weather (Rainfall - Floods) | Yes |
| Wang et al | 2021 | Bacillary Dysentery | Beijing-Tianjin-Hebei, China | Weather (Temperature) | Yes |
| Silva et al | 2021 | Respiratory Disease | Portugal | North African Dust Intrusions | Yes |
| Riancho et al | 2021 | Neurodegenerative diseases (Alzheimer Disease, Parkinsons Disease and Amyotrophic lateral sclerosis) | Spain | Magnetic Fields | No |
| Nili et al | 2021 | Cutaneous Leishmaniasis | Isfahan, Iran | Weather (Precipitation, Temperature, and Relative Humidity) | No |
| Ngo et al | 2021 | Acute Lower Respiratory Infection | Ho Chi Minh City, Vietnam | Weather (Temperature) | Yes |
| Molina-Gómez  et al | 2021 | Respiratory Disease | Kennedy, Bogotá | Air Pollutants | Yes |
| Meng et al | 2021 | Dengue | Guangzhou, China | Weather (Precipitation) | Yes |
| Martinaitiene and Raskauskiene | 2021 | Coronary Artery Disease | Kaunas, Lithuania | Weather (Temperature) | Yes |
| Lindner-Cendrowska and Bröde | 2021 | Influenza | Warsaw, Poland | Universal Thermal Climate Index (UTCI) and Air Pollutants | Yes |
| Lei et al | 2021 | Asthma | Shanghai, China | Weather (Temperature) | Yes |
| Jahan et al | 2021 | Schizophrenia | Queensland, Australia | Seasons | No |
| Gutierrez | 2021 | Leptospirosis | Colombia | Weather (Temperature and Rainfall) | Yes |
| Fdez-Arróyabe et al | 2021 | Influenza | Spain | Circulation Weather | No |
| Dong et al | 2021 | Respiratory Disease | Lanzhou, China | Air Pollutants | Yes |
| Cheng et al | 2021 | Dengue | Guangzhou, China | Weather (Temperature, Rainfall and Humidity) | Yes |
| Chaturrvedi and Dwivedi | 2021 | Malaria | India | Seasonal Temperature | Yes |
| Huang et al | 2022 | Rheumatoid Arthritis | Anqing, China | Weather (Temperature) | Yes |
| Ma et al | 2022 | Influenza A and B | Shenzhen, China | Weather (Temperature, Relative Humidity and Wind Velocity) | Yes |
| Vaičiulis et al | 2022 | Stroke | Kaunas, Lithuania | El Nino Southern Oscillation | Yes |
